# Gut integrity and duodenal enteropathogen burden in undernourished children with environmental enteric dysfunction

**DOI:** 10.1371/journal.pntd.0009584

**Published:** 2021-07-15

**Authors:** Zehra Jamil, Najeeha Talat Iqbal, Romana Idress, Zubair Ahmed, Kamran Sadiq, Indika Mallawaarachchi, Junaid Iqbal, Sana Syed, Aneeta Hotwani, Furqan Kabir, Kumail Ahmed, Sheraz Ahmed, Fayaz Umrani, Jennie Z. Ma, Fatima Aziz, Adil Kalam, Sean R. Moore, Syed Asad Ali

**Affiliations:** 1 Department of Biological and Biomedical Sciences, Aga Khan University, Karachi, Pakistan; 2 Department of Pediatrics and Child Health, Aga Khan University, Karachi, Pakistan; 3 Department of Pathology & Laboratory Medicine, Aga Khan University, Karachi, Pakistan; 4 Department of Public Health Sciences, University of Virginia, Charlottesville, Virginia, United States of America; 5 Division of Pediatric Gastroenterology, Hepatology, and Nutrition; Department of Pediatrics, University of Virginia, Charlottesville, Virginia, United States of America; Saudi Ministry of Health, SAUDI ARABIA

## Abstract

Environmental enteric dysfunction (EED) is a subclinical condition of intestinal inflammation, barrier dysfunction and malabsorption associated with growth faltering in children living in poverty. This study explores association of altered duodenal permeability (lactulose, rhamnose and their ratio) with higher burden of enteropathogen in the duodenal aspirate, altered histopathological findings and higher morbidity (diarrhea) that is collectively associated with linear growth faltering in children living in EED endemic setting. In a longitudinal birth cohort, 51 controls (WHZ > 0, HAZ > −1.0) and 63 cases (WHZ< -2.0, refractory to nutritional intervention) were recruited. Anthropometry and morbidity were recorded on monthly bases up to 24 months of age. Dual sugar assay of urine collected after oral administration of lactulose and rhamnose was assessed in 96 children from both the groups. Duodenal histopathology (n = 63) and enteropathogen analysis of aspirate via Taqman array card (n = 60) was assessed in only cases. *Giardia* was the most frequent pathogen and was associated with raised L:R ratio (p = 0.068). Gastric microscopy was more sensitive than duodenal aspirate in *H*. *pylori* detection. Microscopically confirmed *H*. *pylori* negatively correlated with HAZ at 24 months (r = −0.313, p = 0.013). Regarding histopathological parameters, goblet cell reduction significantly correlated with decline in dual sugar excretion (p< 0.05). Between cases and controls, there were no significant differences in the median (25^th^, 75^th^ percentile) of urinary concentrations (μg/ml) of lactulose [27.0 (11.50, 59.50) for cases vs. 38.0 (12.0, 61.0) for controls], rhamnose [66.0 (28.0, 178.0) vs. 86.5 (29.5, 190.5)] and L:R ratio [0.47 (0.24, 0.90) vs. 0.51 (0.31, 0.71)] respectively. In multivariable regression model, 31% of variability in HAZ at 24 months of age among cases and controls was explained by final model including dual sugars. In conclusion, enteropathogen burden is associated with altered histopathological features and intestinal permeability. In cases and controls living in settings of endemic enteropathy, intestinal permeability test may predict linear growth. However, for adoption as a screening tool for EED, further validation is required due to its complex intestinal pathophysiology.

## Author summary

EED is a subclinical condition of compromised gut integrity secondary to frequent and repeated exposure to enteropathogens in global settings with a high prevalence of undernutrition. In this study, we reported association of gut mucosal architecture with a dual sugar intestinal permeability assay (lactulose-rhamnose) in Pakistani children. In the presence of duodenal enteropathogens, features such as chronic inflammation, intra-epithelial lymphocytosis, enterocyte injury and Paneth cell reduction were consistently observed. When comparing undernourished cases and controls living in the same setting, we found urinary excretion of the sugars was similar among groups; however, variability in HAZ among children at 24 months was partially explained by a model that includes excretion values.

## Introduction

Environmental enteric dysfunction (EED) is a subclinical condition of intestinal inflammation, barrier dysfunction and malabsorption associated with growth faltering in children living in global poverty [1,2]. It is hypothesized that children at risk of EED are frequently exposed to enteropathogen by the fecal-oral route and environmental toxins. In the absence of food security, these insults bring about inflammatory changes in the small intestine during early periods of life or infancy [3]. As a consequence of persistent inflammation and damage to gut epithelium, valuable energy resources is expended [4].

Currently, intestinal biopsies are being assessed across global sites in Asia and Africa to devise a diagnostic criterion for EED using features similar to those for Marsh Scoring of celiac disease [5,6]. In adults, Kelly *et al*. demonstrated defects in the duodenal epithelial lining that led to diminished barrier integrity and associated microbial translocation [7] suggesting an inter-relationship between enteropathogen burden and intestinal permeability. In respect to children, they are maximally prone to stunting in the absence of adequate nutrition, altered gut permeability and enteropathogen associated high morbidity [8,9]. Although microscopy of the gut tissue is the gold standard for exploration of enteropathies, biopsy-based studies in children are extremely challenging in low-income countries. Non-invasive tests for detecting impairment of barrier integrity as a screening tool for EED remains an active area of research. Intestinal permeability tests utilize sugars of different molecular weight (most commonly a monosaccharide coupled with a disaccharide) to differentiate permeability from loss of absorptive surface area across the gut lumen as both the processes overlap and impact gut homeostasis [10,11]. The lactulose: mannitol (L:M) assay has been extensively utilized in the past in various gut disorders but lately, researchers observed endogenous mannitol in approximately 30% of the study participants [12]. Due to this potential limitation and longer timing of urine collection, lactulose: rhamnose (L:R) test is being validated in multiple field settings in place of conventional L:M test. Faubion *et al*. documented raised levels of L:R ratio in Peru and Zambian cohorts of children where EED is endemic while reporting normative values from a US cohort [13]. However, normative values of healthy children living in similar EED endemic settings were not reported. In terms of association between dual sugar tests and histopathological findings on the biopsy, higher L:R ratio has been associated with decreased villous length and epithelial surface area recorded on oriented gut tissues from Zambian adults [14].

Therefore, in children at risk of EED, there was a need to explore the effect of enteropathogen burden on the gut architecture and its absorptive capacity. In this study we hypothesized that a higher burden of enteropathogen in the duodenal aspirate is associated with altered histopathological findings, higher morbidity (diarrhea) and altered duodenal permeability (L, R and L:R ratio) that is collectively associated with linear growth retardation. The aim was to assess the strength of dual sugar assay as a noninvasive test to predict intestinal function in the presence of enteropathogens as they persistently contribute to malnutrition and stunting.

## Methods

### Ethics statement

The ethical approval for this study was granted by the Ethical Review Committee of the Aga Khan University (3836-Ped-ERC-15). For the study participants, written informed consent was obtained from the parents of the participating children.

### Study design & recruitment

**Fig 1:**
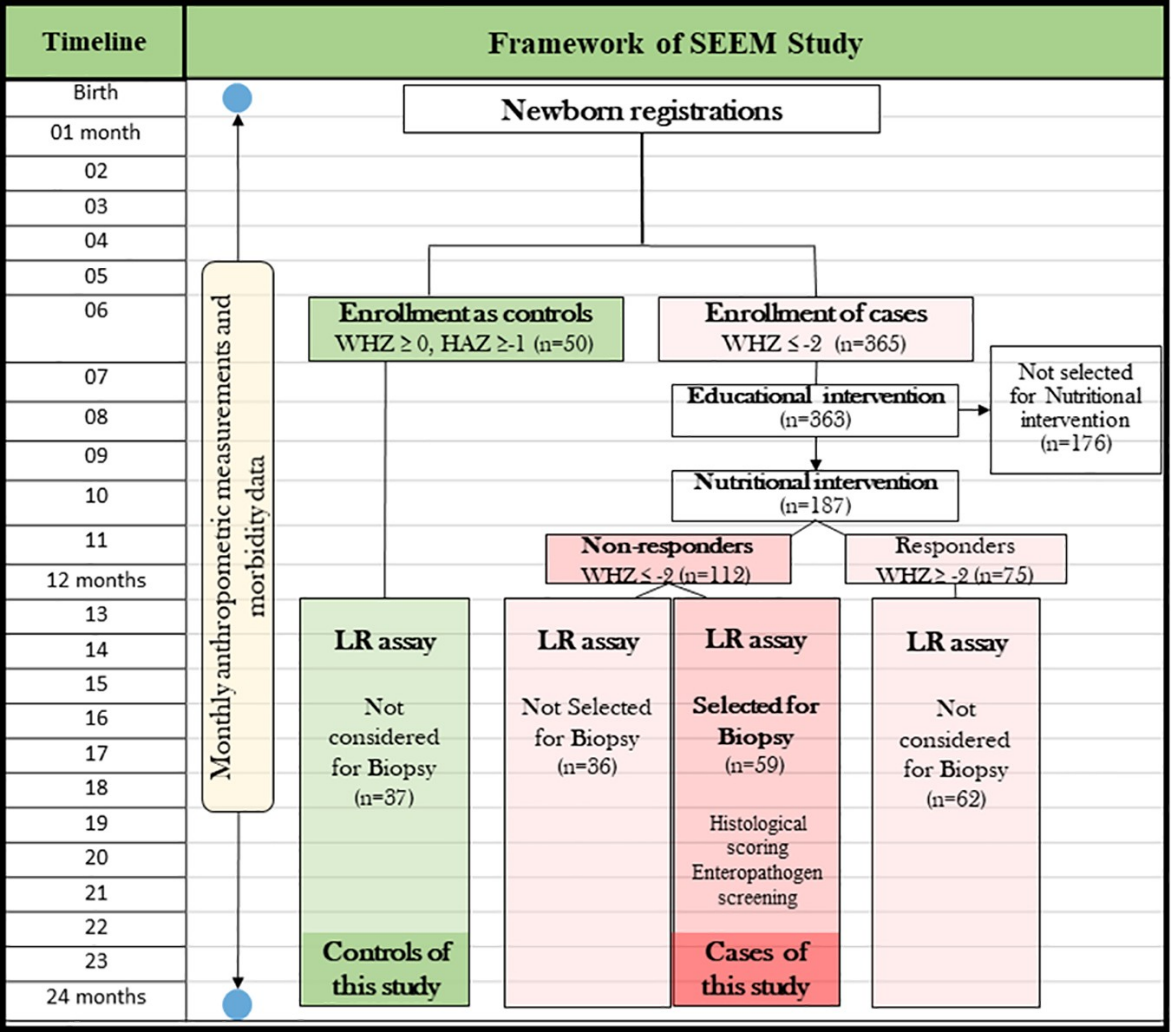
Graphical representation of SEEM study participants and selection of cases with wasting undernutrition refractory to RUST intervention.

This case-control study is a part of a larger community-based intervention study called “Study of environmental enteropathy and malnutrition (SEEM), Pakistan” [6]. In the SEEM study, a birth cohort of 50 newborns was followed for six months and recruited as controls based on at least 2 consecutive months of weight for height Z score (WHZ) > 0 and height for age Z score (HAZ) > −1.0 between birth and 6 months of age. Another cohort of 363 children were followed from birth to 6 months with moderate to severe malnutrition (WHZ < − 2.0) and were recruited as cases for the SEEM study based on any two consecutive WHZ < −2.0 between birth to 6 months. Educational intervention was provided to the parents of all the cases. Of these, 187 (as mentioned in Fig 1) were selected for nutritional intervention at nine months of age as their WHZ remained below -2.0. After administration of Ready to use Supplementary Food (RUSF), all the kids were re-assessed on anthropometry at least one week after completion of the intervention. No improvement was seen in the anthropometric parameters of 112 children (WHZ < -2.0) and were evaluated for endoscopy fitness by two physicians independently (AA and KS). Among these, 24 kids were excluded due to refusal of parental consent, 11 kids were not selected due to co-morbidities, 9 of them crossed the age of 24 months, 3 kids did not complete the nutrition intervention properly, 1 case migrated while 1 child passed away. The remaining 63 children were selected on the bases of clinical evaluation and consent from their parents. These children with wasting undernutrition (WHZ<-2.0) refractory to RUST intervention constitute cases for this study and underwent upper gastrointestinal endoscopy for exploration of treatable causes of malnutrition. Of these, nutritional intervention was extended beyond two months for ten cases due to their deteriorating anthropometry.

In this study, dual sugar assay was assessed in controls and cases that included those who received educational intervention only, educational & nutritional intervention responders and educational & nutritional intervention non-responders (Fig 1). On the other hand, duodenal biopsy evaluation and aspirate analysis were assessed in the undernourished cases who were refractory to nutritional intervention and were selected for endoscopy.

### Administration of lactulose: rhamnose tests

The dual sugar assay of intestinal permeability was successfully assessed in 37 controls, 105 cases who received educational intervention only, 62 responders of nutritional intervention, 36 non-responders of nutritional intervention who were not selected for endoscopy and 59 non-responders who underwent endoscopy. The LR assay was performed between the ages of 10 and 23 months and, in case of active diarrhea, it was postponed. The clinical protocol for saccharide administration was as follows. The reagents were obtained from Sigma Aldrich (St. Louis, US) and Tokyo Chemical Industry (Japan). The oral solution of sugars was prepared by combining 300mg of monosaccharides (L-rhamnose) and 1500mg of disaccharides (lactulose) in 15ml sterile water. Mothers were instructed to give milk or water to the child at least one hour prior to administration of sugar dose. After a fasting of one hour, the community health worker (study personnel) administered the solution over a period of few min to the infants.

For pre-dose urine collection, the urine bag was placed on the children prior to the intake of oral sugar solution, however due to hot weather, the collection of pre-dose urine delayed the collection of urine samples after administration of the sugars. Hence, this was discontinued after collection of 4 urine samples of healthy infants and 9 samples of cases.

### Post-dose urine collection protocol

The pediatric urine bags were placed on the children 20 minutes after administration of the sugar solution. Oral intake of liquids was encouraged to promote production of the urine. The urine was collected over the subsequent 60 minutes as the first sample. In case if no urine was produced as the first sample at 60 minutes, the collection was continued but no longer than 90 minutes. A new urine bag was placed for the collection of the second sample and the urine was collected at 120 minutes post-sugar administration. Participants who failed to urinate during the allocated collection time for their first post-dose sample, were constituted as test failure. The urine samples were transferred to labeled aliquots and stored at -80°C. Frozen aliquots were shipped on dry ice to the Faubion Lab at Mayo Clinic for quantification by HPLC [13].

### Histopathology scoring

Sixty-three non-responders to nutritional intervention underwent upper GI endoscopy (performed by KS) at the Aga Khan University Hospital (AKUH), Karachi. The age of the children at the time of endoscopy ranged between 12.5 months to 24 months. There was no report of any adverse event during or after the procedure. For histopathological workup, two biopsies were collected from the gastric antrum while two to three biopsies were collected from second part of the duodenum (n = 63). In some cases, single biopsy was also collected from sigmoid (n = 17) and rectum (n = 20) respectively. All the specimens were immediately placed in 10% formalin buffer solution and separately embedded in the paraffin block later to be stained by hematoxylin and eosin (H &E) stain. The biopsy tissues were processed at the clinical laboratory and graded by two independent pathologists at AKU who were blinded to enteropathogen and clinical data. The duodenal biopsies were graded as per the EED scoring index criteria. This criterion has been developed by gastrointestinal pathologists with input from members of EED Biopsy Initiative Consortium and consists of eleven parameters [15]. As maximum of three slides were available for a single biopsy taken from D2/D3 part of duodenum, the pathologists (RI and ZA) scored the specimen as a subjective average across the tissue sections on the three slides. The microscopic tissue of every case was scored on the bases of eleven features with an aggregate score of 37 [15].

### Enteropathogen analysis of duodenal aspirate

The duodenal aspirate was collected during endoscopy from the second portion of the duodenum after infusion of 10 to 25ml normal saline solution via sterile catheter. The fluid sample was placed in 1.5ml aliquot and stored at −80°C until further workup. To explore the enteric pathogen panel that included viruses, bacteria, and protozoa, Taqman Array Card (TAC) was customized to detect microbial pathogens in the duodenal aspirate (manufactured by Life Technologies, Thermo fisher, US). This card was customized to detect common enteropathogens as listed in the S7 Table at the Houpt Laboratory, University of Virginia, Charlottesville, VA [6].

### Statistical analysis

Normally distributed quantitative variables were summarized as mean ± standard deviation (SD) and compared between cases and controls using two-sample t-test. Skewed distributed quantitative variables were summarized as median (25^th^, 75^th^ percentile) and compared between cases and controls using the Mann-Whitney U test. Analysis of variance (ANOVA) was used to compare normally distributed quantitative variables among multiple groups. Kruskal-Wallis test was used to compare skewed distributed quantitative variables among multiple groups. Categorical variables were summarized as frequency as well as percentages and compared cases with controls using Fisher’s exact test. Pearson correlation was used to explore measure the strength of association between two normally distributed quantitative variables while Spearman correlation was used for associations between skewed quantitative variables.

Univariate and multivariable linear regression was used to evaluate the effect of risk factors on the HAZ at 24 months. Initial HAZ, initial WAZ, newborn gender, gestational age, diarrhea episodes, and log-transformed lactulose and rhamnose were added as the possible confounders. Interaction between predictors was also considered. Backward stepwise regression with probability of 0.10 for both entry and removal was performed to obtain the final model. Model assumptions were validated using studentized residuals. One child was identified as an outlier based on the influential index and excluded from the analysis.

All the analysis was conducted using STATA Statistical software 15. StataCorp. 2017. College Station, TX: StataCorp LLC. Figures were produced in R version 4.0.2 (R Core Team, 2020) using the package ggplot2.

## Results

Descriptive statistics of our study population are summarized in Table 1. Anthropometry parameters during the follow-up period were significantly lower in cases compared to the controls. The cases comprised of 60.1% males while they constituted 52% of the controls. Females responded better to the educational intervention, yet it was statistically insignificant (p = 0.16) Average gestational age was significantly different between cases and controls (38.62 ± 0.97 vs 38.90 ± 0.68, p = 0.021). Median diarrhea episodes per year for the first 24 months were highest in cases who were selected for endoscopy (p = 0.20).

**Table 1:** Demographics of the study cohort.

Factor	Controls (n = 51)	Cases (n = 365)				p value (controls vs cases selected for endoscopy)
		Educational intervention only (n = 178)	Educational & nutritional intervention			
			Nutritional Intervention Responders (n = 75)	Nutritional Intervention Non-responders		
				Not selected for endoscopy (n = 49)	Selected for endoscopy (n = 63)	
Newborn gender						0.16
Male	26 (52.0%)	102 (46.36%)	49 (41.53%)	25 (36.23%)	44 (63.77%)	
Female	24 (48.0%)	74 (51.75%)	26 (37.68%)	24 (55.81%)	19 (41.19%)	
Gestational age (wks)	38.90 ± 0.68	38.87 ± 0.80	38.49 ± 1.45	38.48 ± 1.47	38.62 ± 0.97	0.021
WHZ at 6 months	0.84 ± 0.93	-1.97 ± 0.77	-2.63 ± 0.70	-2.70 ± 0.81	-2.89 ± 0.82	<0.001
WAZ at 6 months	0.11 ± 0.93	-2.91 ± 1.14	-3.40 ± 1.16	-3.90 ± 1.17	-3.56 ± 1.04	<0.001
HAZ at 0–1 months	-0.88 ± 0.96	-1.54 ± 1.18	-1.69 ± 1.31	-2.47 ± 1.33	-2.11 ± 1.17	<0.001
HAZ at 6 months	-0.75 ± 0.93	-2.17 ± 1.35	-2.40 ± 1.48	-3.04 ± 1.39	-2.28 ± 1.14	<0.001
HAZ at 12 months	-1.05 ± 1.18	-2.20 ± 1.17	-2.53 ± 1.22	-3.10 ± 1.39	-2.83 ± 1.10	<0.001
HAZ at 24 months	-1.44 ± 1.15	-2.21 ± 1.03	-2.38 ± 1.13	-3.15 ± 1.39	-2.90 ± 1.14	<0.001
Diarrhea episodes per year	8.98 (6.36, 11.82)	9.46 (5.75, 13.89)	8.89 (5.46, 12.23)	9.51 (6.62, 14.60)	11.03 (7.71, 15.21)	0.20
Age (months) at the time of endoscopy					19.15 ± 3.67	
Age (months) at the time of dual sugar assay	14.19 ± 2.02	13.89 ± 1.94	13.45 ± 1.45	14.00 ± 1.55	14.17 ± 2.48	0.23
Lactulose (μg/ml)	38.0 (12.0, 61.0)	28.0 (12.0, 55.0)	30.5 (13.0, 58.5)	31.0 (14.5, 59.0)	27.0 (11.5, 59.5)	0.92
Rhamnose (μg/ml)	86.5 (29.5, 190.5)	66.0 (28.0, 145.0)	77.0 (33.0, 144.0)	65.0 (23.0, 139.0)	60.0 (28.0, 178.0)	0.71
LR ratio	0.51 (0.31, 0.71)	0.50 (0.29, 0.81)	0.39 (0.27, 0.58)	0.47 (0.29, 0.97)	0.47 (0.24, 0.90)	0.47

Whereas WHZ is weight for height z score, WAZ is weight for age z score, HAZ is height for age z score. Diarrheal episode per year is defined as a minimum of 2 days with diarrhea followed by at least 2 diarrhea-free days per year.

Data are expressed as mean ± SD or median (25^th^, 75^th^ percentile) for continuous variables and frequency (percentage) for categorical variables.

### Intestinal permeability tests in children at risk of EED

**Fig 2:**
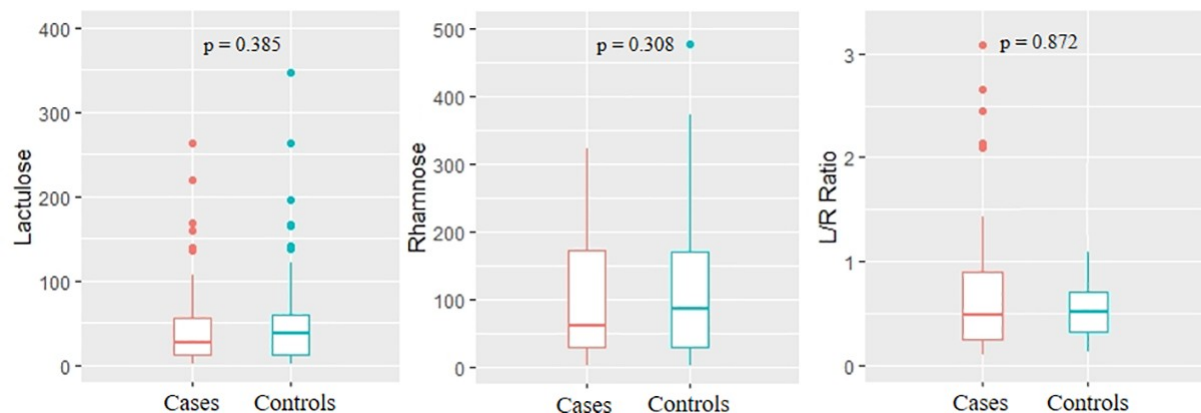
Comparison of urinary excretion of lactulose and rhamnose following oral administration in cases (n = 59) and controls (n = 37). Lactulose and rhamnose are expressed as medians (q1,q3).

A non-significant difference was observed in the levels of lactulose [median (25^th^, 75^th^ percentiles)] between cases and controls [27.0 (11.50, 59.50) and 38.0 (12.0, 61.0)] respectively. Permeability to rhamnose was reported as 66.0 (28.0, 178.0) and 86.5 (29.5, 190.5) as shown in Fig 2. Similarly, no difference was seen in the L:R ratios of cases [0.47 (0.24, 0.90)] and controls [0.51 (0.31, 0.71)].

Next, we assessed the ability of dual sugars to predict linear growth in cases and controls. Table 2 reports the final model for HAZ at 24 months as an outcome (n = 96). After multivariable adjustment, one unit increase of log lactulose was associated with a decrease in HAZ score by 0.35 units while one unit increase of log rhamnose was associated with an increased HAZ by 0.32 units. About 31% of the variability in HAZ among children was explained by the final model including lactulose and rhamnose excretion.

**Table 2:** Multivariable linear regression model including variables significant at 10% level to predict 24 months HAZ in cases and controls.

	Full Model	p value	Final Model	p value
HAZ (0–1 mo)	0.72 (0.362, 1.078)	<0.001	0.611 (0.414, 0.809)	<0.001
WAZ (0–1 mo)	-0.138 (-0.465, 0.19)	0.406		
Gestational age	0.082 (-0.198, 0.362)	0.564		
log lactulose	-0.352 (-0.712, 0.008)	0.055	-0.350 (-0.683, -0.018)	0.039
log rhamnose	0.321 (-0.017, 0.658)	0.062	0.319 (0.013, 0.625)	0.041
Gender: Female	0.616 (0.128, 1.105)	0.014	0.592 (0.116, 1.068)	0.015
Mean diarrhea prevalence	-0.001 (-0.007, 0.004)	0.595		

Note: Model: 24m HAZ = β0+β1 HAZ at enrollment+β2 WAZ at enrollment + β3 log lactulose+ β4 log rhamnose + β5 gender (female). Adjusted R square of the final model is 31%.

### Histopathological features of duodenal biopsies of children at risk of EED

**Fig 3:**
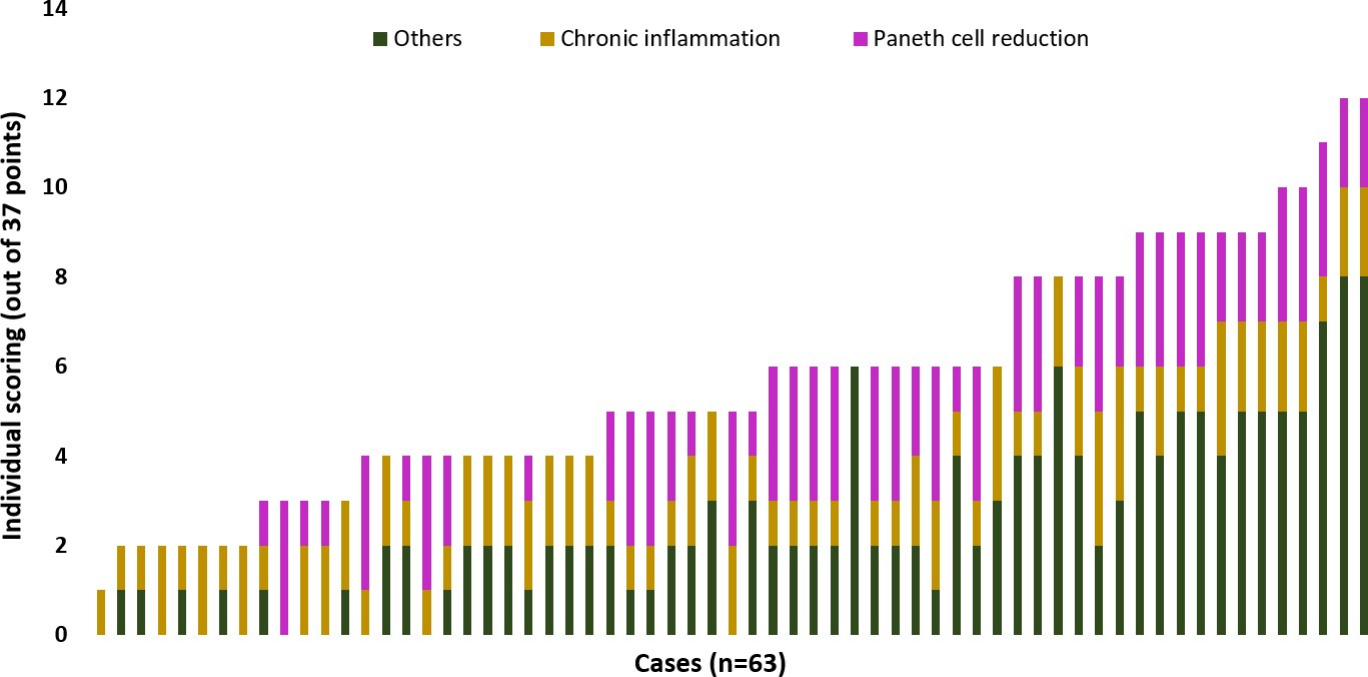
Histopathological scoring of individual cases (n = 63). Accumulative scores of eleven parameters graded as per the EED scoring index developed by gastrointestinal pathologists with input from members of the EED Biopsy Initiative Consortium. Out of 63 cases, two biopsy specimens scored 12 out of 37 while one scored 1 out of 37. Chronic inflammation and reduction in Paneth cell density were the most consistent features.

**Fig 4:**
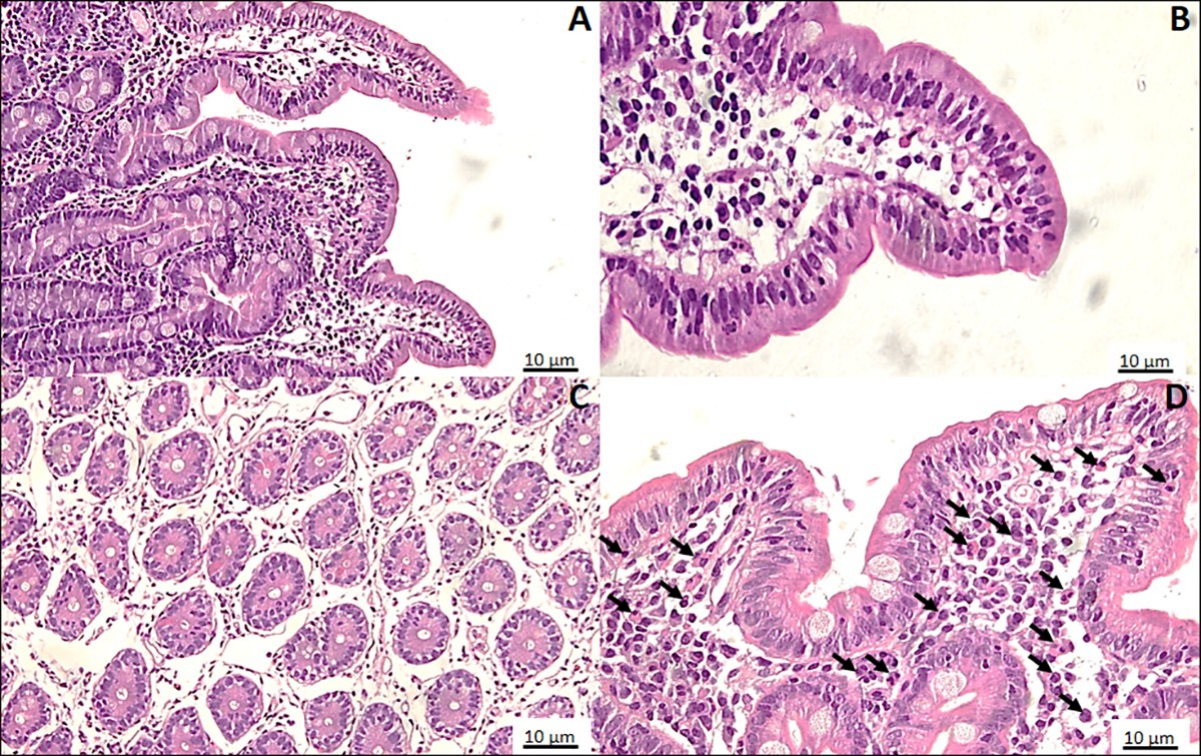
H &E photomicrographs of duodenal biopsies. (**A)** Tissue obtained from a child who scored 4 out of 37 on the EED severity scale, with normal goblet cell density (100x). (**B)** Severe reduction in the goblet cells in a blunted villous of a child with a score of 12 (400x). (**C)** loss of Paneth cells seen in the duodenal crypts (200x). (**D)** Chronic inflammation characterized by polymorphonuclear leukocytes marked as black arrows (400x).

Fig 3 represents the histopathological scores of duodenal specimens against eleven parameters with a total of 37 points. The median histopathological score was 5, where thirty-three children scored between 1 and 5 while thirty children scored between 6 and 12. No difference was seen in the anthropometric measurements of low and high scorers. Chronic inflammation was a consistent feature on most of the microscopic tissues, followed by reduction in the Paneth cells (Figs 4 and S1). Among all the histopathological parameters, goblet cell reduction was significantly associated with a decline in urinary excretion of lactulose and rhamnose (S1 Table). Microscopy of gastric, sigmoidal and rectal tissues have been summarized in S8 Table.

### Association of enteropathogen with gut permeability and histopathological features

**Fig 5:**
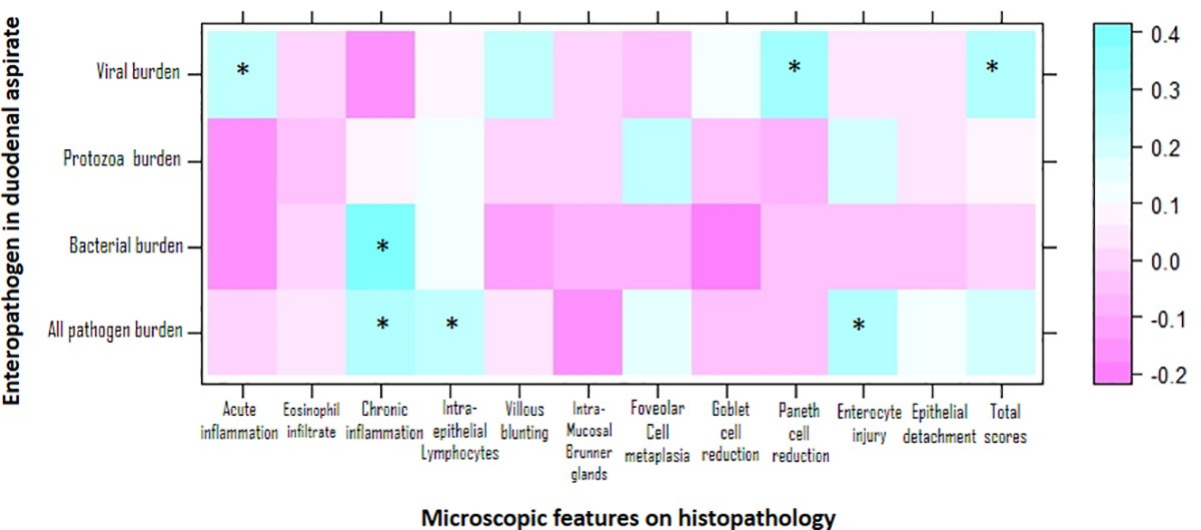
Correlation coefficient matrix of pathogens in the duodenal aspirate with the histopathological features in cases (n = 60). Values are Spearman’s correlation coefficients. *p < 0.05. The following were studied as continuous variable: presence of any pathogens, bacteria, protozoa, viruses and all histopathological features.

Table 3 presents association of pathogen in the duodenal aspirates infected with different subtypes. No difference was noted between the mean HAZ in children with or without a particular enteropathogen. Similar diarrheal burdens and L:R ratio were seen in children with or without pathogens. Positive correlations were observed between acute neutrophilic infiltration with viruses; chronic inflammation with presence of any pathogen and bacteria; intra-epithelial lymphocytes with presence of any pathogens, Paneth cell density reduction and total scores with viruses; and enterocyte injury with presence of any pathogens (Fig 5). Additionally, association between the presence of any pathogen, bacteria, protozoa or viruses in the duodenal aspirate has been provided in S7 Table.

**Table 3:** Summary of the enteropathogen in the duodenal aspirate and its association with HAZ, morbidity and dual sugar urinary excretion in cases.

	Enteropathogens in duodenal aspirate (n = 60)											
	Any pathogens			Bacteria			Protozoa			Viruses		
	Yes	No	p	Yes	No	p	Yes	No	p	Yes	No	p
N (%)	51 (85%)	9 (15%)		12 (20%)	48 (80%)		40 (67%)	20 (33%)		9 (15%)	51 (85%)	
HAZ 24mo	-2.79 ±1.11	-3.19 ±1.24	0.34	-3.07 ±1.25	-2.80 ±1.10	0.45	-2.87 ±1.15	-2.82 ±1.12	0.87	-2.29 ±1.13	-2.94 ±1.11	0.13
WHZ 24 mo	-1.86 ±0.80	-2.27 ±0.72	0.69	-2.01 ±0.63	-1.88 ±0.83	0.29	-1.71 ±0.79	-2.37 ±0.58	0.24	-1.90 ±0.74	-1.92 ±0.81	0.82
Diarrhea episodes / year	11.5 (7.70, 15.3)	9.2 (7.80, 13.4)	0.37	12.05 (10.2, 14.9)	10.9 (7.40, 15.4)	0.30	11.4 (8.70, 15.3)	10.9 (6.70, 15.5)	0.59	14.4 (4.90, 18.8)	11.0 (7.90, 14.5)	0.57
Lactulose μg/ml	26.0 (12.0, 59.5)	19.0 (2.6, 36.0)	0.32	19.0 (12.0, 66.0)	26.0 (10.0, 48.0)	0.90	26.0 (12.0, 66.0)	20.0 (10.0, 36.0)	0.37	23.5 (14.9, 38.0)	25.0 (11.0, 53.0)	0.85
Rhamnose μg/ml	54.0 (29.0, 178.0)	30.0 (15.0, 233.0)	0.73	29.0 (11.0, 178.0)	60.0 (30.0, 185.0)	0.34	53.0 (22.0, 185.0)	56.0 (29.0, 178.0)	0.84	82.0 (44.0, 203.0)	52.0 (22.0, 165.0)	0.19
L:R ratio	0.48 (0.25, 0.88)	0.35 (0.12,0.80)	0.17	0.52 (0.25, 1.10)	0.41 (0.23, 0.80)	0.37	0.48 (0.26,0.88)	0.35 (0.16, 0.80)	0.11	0.24 (0.19, 0.42)	0.48 (0.26, 0.88)	0.09

Notes: “Yes” = infection with 1 or more pathogens of the specified category. “No” = no infections of the specified category. HAZ 24 = Height-for-age Z score at the 24th months of life. None of the differences in HAZ score between children infected or not infected with pathogens of a given category were significant. p < 0.05 was considered as significant.

Data are expressed as mean ± SD or median (25^th^, 75^th^ percentile) for continuous variables and frequency (percentage) for categorical variables.

### Effect of selected pathogens on the gut permeability and histology

Next we explored the effect of *Giardia*, *Campylobacter species (C*. *jejuni and C*. *coli) and Helicobacter pylori (H*. *pylori)* in the duodenal aspirate (S2 Table). *Giardia* was the most common organism detected on the TAC panel (n = 38) while on microscopy, 26 samples confirmed its presence (S3 Table). Although there was no difference in diarrheal episodes, giardiasis was non-significantly associated with an increased L:R ratio (p = 0.078). This finding was consistent with giardiasis confirmed on microscopy (p = 0.068).

Regarding *H*. *pylori*, gastric microscopy was more sensitive than TAC in detection of the pathogen as 7 aspirates and 29 H & E stained microscopic slides confirmed its presence (S2 and S3 Tables). These cases reported non-significantly higher L:R ratio. While evaluating the relationship with the histopathological features of duodenal biopsies, giardiasis showed no association with any of the parameters. In the context of linear growth, a lower HAZ at 24 months was observed in microscopically confirmed *H*. *pylori* (−3.29 vs −2.56, p = 0.014) and *Giardia* cases (−3.17 vs −2.71, p = 0.091), yet this association was not observed in cases confirmed on duodenal aspirate analysis.

## Discussions

The prospective birth cohort in the SEEM study provides a unique opportunity to explore complex relationship between intestinal permeability, duodenal histopathological features, enteropathogen burden, diarrhea and growth in undernourished children. In this study, chronic inflammation may have been consistently seen and pathogens were detected in 85% of children, but neither chronic inflammation nor pathogens were significantly associated with permeability assays in the data presented. Intestinal permeability as reflected by urinary lactulose and rhamnose secretion was similar in the cases and controls however, it accounted for 31% of variation in HAZ at 24months while controlling for gender and first month anthropometry. In the context of malnutrition, this is the first study which reports dual sugar urinary excretion levels in undernourished cases and controls living in the same settings of endemic enteropathy.

Regarding association of intestinal permeability and impaired linear growth, studies report higher L:R ratio in children living in EED endemic settings while comparing them to the US healthy controls [13]. The literature consistently suggests effect of epidemiological settings on the recovery of the dual sugar, we compared assay in two cohorts living in the same setting [16]. Although there was no significant difference in the L:R ratio, rhamnose malabsorption in cases seemed to be the chief contributor to the ratio reflecting villus blunting. Amongst the controls, those twenty kids who consistently reported a decent linear growth up to twenty-four months of age, had noticeably higher rhamnose than the cases (S4 and S5 Tables). However, these findings were statistically non-significant owing to a smaller sample size. Thus, rhamnose may have the potential to be explored as a marker to indicate gut health in children at risk of developing stunted growth. On the other hand, lactulose levels were marginally higher in controls while no difference was observe in the L:R ratio. This can be explained by the fact that alterations in gut barrier function are expected in even asymptomatic healthy children as they are equally exposed to varying degrees of chronic inflammation due to pathogen burden, oro-fecal contamination and exposure to environmental toxins [6]. In our study, we did not observe age-dependent or gender-dependent effect on gut-permeability measurements (S6 Table).

Moreover, impaired intestinal permeability seemed to explain variation in the HAZ at the age of 24 months while controlling for gender and anthropometric measures during the first month of life. In this study, urine collection was strictly limited to within 90 minutes of oral sugar load as the literature suggests that delayed collection of urine is more reflective of colonic absorption [17]. Therefore, in the regression analysis, gut permeability reflecting small intestinal absorption accounted for 31% of variation in HAZ. We propose the worth of gut permeability test in prediction of growth faltering by 24 months of age however, it should be interpreted cautiously while administrating as a screening tool for EED due to its complex intestinal pathophysiology where gut permeability is one of the several influencers that collectively leads to malnutrition.

As we investigated the effect of pathogens in duodenal aspirate on linear growth, no association was seen with HAZ at 24 months. Previous studies have reported a substantial negative association with pathogens in the stool samples particularly *Shigella*, *Enteroaggregative E*.*coli*, *Campylobacter* and *Giardia* [18,19]. In the context of histopathological features, enteropathogens significantly correlated with chronic inflammation, intra-epithelial lymphocytosis and enterocyte injury. Although we did not find association with villous blunting, a recently published study proposed that in EED, reduced villous surface was a compensatory mechanism to minimize microbial translocation yet, at the cost of impaired growth [20]. Our group has previously reported greater magnitude of lamina propria T-cell infiltrates and intraepithelial lymphocytes in EED cases as compared to celiac disease in the presence of microbial overgrowth in duodenal aspirate [5]. In an EED endemic setting, we observed that majority of children whose undernutrition merited an endoscopic evaluation had at least one pathogen in their duodenal aspirate (85%) with indeterminate effects on their linear growth, diarrheal burden and intestinal permeability.

*Giardia* was the most frequent pathogen in our study (38/60 in duodenal aspirates; 26/63 on H&E slides). Giardiasis is a major cause of diarrheal diseases in young children; however, there are conflicting reports regarding its protective effect on subsequent diarrhea. Our results support findings from the MAL-ED birth cohort, where no protective effect was observed on the diarrheal prevalence [19]. Detection of *Giardia* on microscopy correlated with HAZ at 24 months (p = 0.091) supporting previous studies that linked giardiasis with the risk of stunting [21]. Detection of *H*. *pylori* on gastric microscopy was more sensitive than TAC analysis of the aspirates. Multiple studies have shown association between *H*. *pylori* infection and growth in children [22]. However, most of these studies detected pathogens on stool examination, followed by the urea breath test and specific serum IgG antibodies. We observed significant correlation of microscopically confirmed *H*. *pylori* and linear growth at 24 months (r = −0.313, p = 0.013). Additionally, it further correlated with *Campylobacter* (r = 0.468, p <0.001) and *Norovirus* (r = 0.358, p = 0.005) suggesting increased risk of co-infections in the presence of altered stomach pH with *H*. *pylori*.

Regarding intestinal permeability between cases with or without enteropathogens, *Giardia* positive cases reported a non-significant increase as reflected by higher L:R ratio (p = 0.078). Of note, lower levels of rhamnose seemed to contribute to this rise suggesting loss of intestinal surface area. These findings are in line with studies that suggest early giardiasis as a contributing factor to intestinal permeability and stunted growth, independent of diarrheal prevalence [19]. On the contrary, *H*. *pylori* was associated with higher lactulose as well as rhamnose in the presence of frequent diarrhea. Raised L:R ratio was also seen in comparison to cases without *H*. *pylori*, yet it was non-significant. This suggests that different pathogens produce their detrimental effects through diverse mechanisms and hence, sole nutritional interventions may not achieve desirable effects. Recently, study published by Robert Y. Chen *et al*. has shed new lights on the relationship between growth faltering and components of small intestinal microbiota in the context of EED supporting therapeutic role of targeting these microbial communities [23].

In reference to histopathological features, reduction in the goblet cell count was significantly associated with a decline in the urinary dual sugars’ excretion (S1 Table). Goblet cell-generated mucins are essential components of the intestinal mucous layer, a major contributor to intestinal barrier function. Previously published data suggest reduced expression of several mucins and regulatory factors in children with EED [11]. Other than goblet cell reduction, no noteworthy association was seen as additional parameters included factors (for example Paneth cell density or lymphocyte infiltrations) that are not linked to gut permeability. Exploration with morphometric analysis of duodenal tissue may provide a better insight in terms of histological associations with intestinal permeability test.

The limitation of this study includes lack of pre-administration urinary levels of dual sugars for normalization of the data. The study site Matiari is a rural area with an average temperature ranging between 100 to 115°F during summers. Collection of pre-dose urinary samples in hot and humid weather hampered with the success rate of collecting post-dose urine samples within allocated time. Secondly, although we recruited controls on the bases of WHZ > 0 by the age of 6 months, a decline in their anthropometric measurements suggested a negative impact of the environment on children that were born healthy. As a result, anthropometric measurements of some of our controls may have dropped below the WHZ cutoff at the time of dual sugar absorption test. Regarding enteropathogen evaluation, samples were collected during upper GI endoscopy where antibiotics were prescribed to kids with diarrhea. This may have affected the presence of pathogens in the duodenal aspirate. Regarding timings of the LR assay and endoscopy, permeability assay was performed before the endoscopy in 48 cases. However, in the remaining 12 cases the assay was performed at least 1 week after the endoscopy either due to active diarrhea or assay failure during the first attempt. Data on histopathological findings and duodenal pathogen burden was only available for the cases. Lastly, we do not have concurrent fecal pathogen data from the lower GI to correlate with upper GI data.

In conclusion, enteropathogen burden in the duodenal aspirate is associated with altered histopathological features and intestinal permeability. Moreover, specific pathogens may increase the risk of stunting at 24 months of age and are also associated with higher intestinal permeability as reflected by raised L:R ratio. Although urinary levels of the sugars were similar in cases and controls living in the same EED endemic setting, the dual sugar gut permeability test may predict linear growth at 24 months of age. For adoption as a screening tool for EED, further validation is required due to its complex intestinal pathophysiology. As multiple factors are involved in linear growth impedance in children at risk of EED, there is a potential role of interventions aiming to restore barrier integrity in order to minimize the risk of developing stunted growth.

## Supporting information

S1 Fig Individual scoring pattern (median IQR) of eleven histology features. The scoring index of epithelial detachment, intra-epithelial lymphocytes and villous blunting was based on a 5-tier grading (scale of 0 to 4) while for the rest of the parameters it was graded on 4-tier categorical values (0 to 3).
(TIF)

S1 Table Spearman correlation coefficient of Lactulose, Rhamnose and L:R ratio with histopathological features (in cases only). (DOCX)

S2 Table Association of *Giardia*, *Helicobacter pylori* and *Campylobacter* in the duodenal aspirates with linear growth, morbidity and intestinal permeability. (DOCX)

S3 Table Association of selected pathogens on microscopy with growth, morbidity and intestinal permeability. (DOCX)

S4 Table Comparison of demographic characteristics of cohorts from Faubion *etal* study [13] (USA, Peru & Zambia) and Pakistani cohorts. (DOCX)

S5 Table Sub-group analysis of dual sugars to gain further insight. (DOCX)

S6 Table Comparison of dual sugar urinary excretion between gender and different age groups. (DOCX)

S7 Table Statistical comparison between each pathogen group vs no pathogen (**A**) and their spearman correlation (**B**).
(DOCX)

S8 Table Microscopic features of gastric, sigmoidal and duodenal biopsies from cases. (DOCX)

S1 Data Summary of TAC analysis of the duodenal aspirate (n = 60). (XLSX)
