# Supplementary figures and images for: Gut integrity and duodenal enteropathogen burden in undernourished children with environmental enteric dysfunction
Source: PLoS Negl Trop Dis. 2021 Jul 15;15(7):e0009584. doi: 10.1371/journal.pntd.0009584 (PMC8352064; doi:10.1371/journal.pntd.0009584)

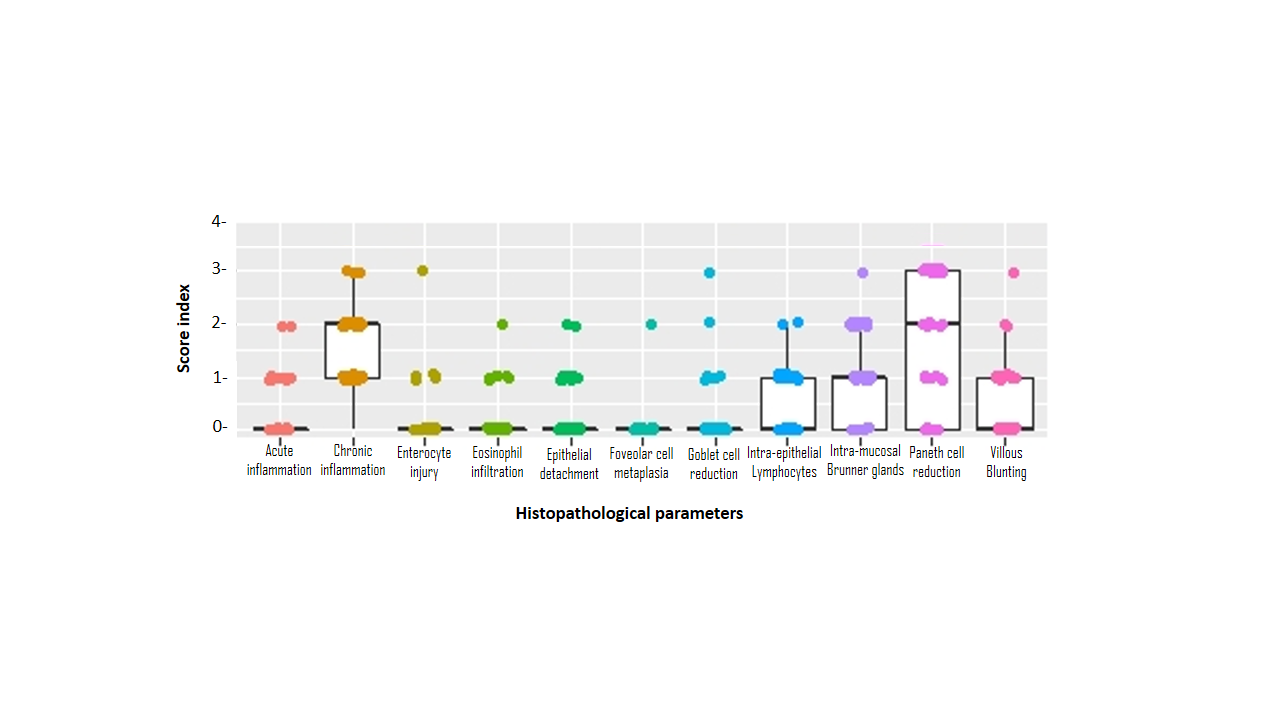

Supplement: S1 Fig — The scoring index of epithelial detachment, intra-epithelial lymphocytes and villous blunting was based on a 5-tier grading (scale of 0 to 4) while for the rest of the parameters it was graded on 4-tier categorical values (0 to 3). (TIF) [file pntd.0009584.s001.tif]
