# Supplementary material for: Gut integrity and duodenal enteropathogen burden in undernourished children with environmental enteric dysfunction
Source: PLoS Negl Trop Dis. 2021 Jul 15;15(7):e0009584. doi: 10.1371/journal.pntd.0009584 (PMC8352064; doi:10.1371/journal.pntd.0009584)
Supplement: S1 Table — (DOCX) [file pntd.0009584.s002.docx]

**S1 Table:** Spearman correlation coefficient of Lactulose, Rhamnose and L:R ratio with histopathological features (in cases only).

| **Histopathology scoring criteria** | **Lactulose** | **Rhamnose** | **L:R ratio** |
| --- | --- | --- | --- |
| Acute infiltration | -0.222 | -0.081 | -0.112 |
| Eosinophil infiltration | 0.179 | 0.09 | 0.054 |
| Chronic inflammation | -0.152 | -0.126 | 0.021 |
| Intra-epithelial lymphocytes | 0.010 | 0.018 | 0.054 |
| Villus blunting | -0.177 | -0.153 | 0.092 |
| Intramucosal Brunner glands | -0.058 | 0.012 | -0.043 |
| Foveolar cell metaplasia | 0.120 | 0.177 | -0.093 |
| Goblet cell reduction | **-0.317 *** | **-0.355 *** | 0.211 |
| Paneth cell reduction | 0.005 | -0.028 | 0.139 |
| Enterocyte injury | -0.012 | 0.035 | -0.043 |
| Epithelial detachment | -0.087 | -0.107 | 0.031 |
| Total score (out of 37) | -0.112 | -0.065 | 0.073 |

Note: Values are Spearman’s correlation coefficients. *p <0.05. The following were studied as continuous variables: Lactulose, Rhamnose, L:R ratio and all histopathological features.
