# Supplementary material for: Gut integrity and duodenal enteropathogen burden in undernourished children with environmental enteric dysfunction
Source: PLoS Negl Trop Dis. 2021 Jul 15;15(7):e0009584. doi: 10.1371/journal.pntd.0009584 (PMC8352064; doi:10.1371/journal.pntd.0009584)
Supplement: S2 Table — (DOCX) [file pntd.0009584.s003.docx]

**S2 Table:** Association of *Giardia, Helicobacter pylori* and *Campylobacter* in the duodenal aspirates with linear growth, morbidity and intestinal permeability.

|  | **Selected enteropathogen in the duodenal aspirate (n=60)** | | | | | | | | |
| --- | --- | --- | --- | --- | --- | --- | --- | --- | --- |
|  | **Giardia** | | | **H. pylori** | | | **Campylobacter pan** | | |
|  | **Yes** | **No** | **p** | **Yes** | **No** | **p** | **Yes** | **No** | **p** |
| N (%) | 38 (63.3%) | 22 (36.7%) |  | 7 (11.7%) | 53 (88.3%) |  | 9 (15%) | 51 (85.0%) |  |
| HAZ 24mo  Mean (SD) | -2.92 (1.15) | -2.74 (1.10) | 0.56 | -3.24 (1.50) | -2.80 (1.08) | 0.34 | -2.70  (1.06) | -2.88 (1.15) | 0.66 |
| WHZ 24mo mean (SD) | -1.72 (0.81) | -2.29 (0.63) | 0.32 | -2.05 (0.45) | -1.90 (0.83) | 0.10 | -2.18  (0.69) | -1.87 (0.81) | 0.54 |
| Diarrhea episodes/ year Median (IQR) | 11.4 (8.7, 15.2) | 10.9 (6.4, 16.2) | 0.64 | 11.8 (10.0, 15.2) | 11.0 (7.7, 15.4) | 0.68 | 12.3 (10.5, 16.7) | 11.0 (7.2, 15.3) | 0.31 |
| Lactulose µg/ml | 26.0 (12.5, 66.0) | 20.0 (10.0, 36.0) | 0.29 | 59.5 (28.0, 77.0) | 20.0 (9.8, 47.0) | 0.10 | 17.0 (10.6, 40.5) | 27.0 (11.0, 48.0) | 0.52 |
| Rhamnose µg/ml | 53.0 (25.0, 175) | 56.0 (28.5, 205) | 0.76 | 116.0 (29.0, 199) | 52.0 (28.0, 165) | 0.50 | 41.5 (16.6, 127.5) | 56.0 (28.5, 185) | 0.34 |
| L:R ratio | 0.48 (0.26, 0.93) | 0.35 (0.16, 0.82) | 0.078 | 0.70 (0.21, 1.09) | 0.42 (0.24, 0.84) | 0.56 | 0.47 (0.27, 0.93) | 0.42 (0.23, 0.86) | 0.71 |

Notes: “Yes” = infection with 1 or more pathogens of the specified category. “No” = no infections of the specified category. Lactulose, Rhamnose and L:R ratio expressed as medians (q1,q3)
