# Supplementary material for: Gut integrity and duodenal enteropathogen burden in undernourished children with environmental enteric dysfunction
Source: PLoS Negl Trop Dis. 2021 Jul 15;15(7):e0009584. doi: 10.1371/journal.pntd.0009584 (PMC8352064; doi:10.1371/journal.pntd.0009584)
Supplement: S3 Table — (DOCX) [file pntd.0009584.s004.docx]

**S3a Table:** Association of selected pathogens on microscopy with growth, morbidity and intestinal permeability.

|  | **Selected enteropathogen in the histopathology (n=63)** | | | | | |
| --- | --- | --- | --- | --- | --- | --- |
|  | **Giardia** | | | **H. pylori** | | |
|  | **Yes** | **No** | **p** | **Yes** | **No** | **p** |
| N (%) | 26 (41.2%) | 37 (58.8%) |  | 29 (46.03%) | 34 (53.97%) |  |
| HAZ 24mo  Mean (SD) | -3.17 (1.10) | -2.71 (1.14) | 0.091 | -3.29 (1.21) | -2.56 (0.97) | 0.014 |
| WHZ 24mo Mean (SD) | -1.82 (0.80) | -1.99 (0.79) | 0.718 | -1.83 (0.77) | -2.00(0.82) | 0.938 |
| Diarrhea episodes/ year Median (IQR) | 11.3 (8.3, 15.2) | 11.0 (7.1, 14.9) | 0.615 | 11.9 (9.2, 16.4) | 9.9 (6.6, 13.8) | 0.087 |
| Lactulose µg/ml | 35.0 (11.2, 59.5) | 20.0 (11.0, 66.0) | 0.594 | 28.0 (13.5, 74.3) | 24.5 (7.2, 45.5) | 0.335 |
| Rhamnose µg/ml | 52.0 (19.5, 142) | 78.0 (29.8, 189.2) | 0.357 | 81.0 (29.25, 174.8) | 51.0 (22.0, 203.0) | 0.425 |
| L:R ratio | 0.64 (0.37, 1.01) | 0.40 (0.21, 0.85) | 0.068 | 0.40 (0.22, 0.96) | 0.48 (0.26, 0.90) | 0.704 |

Notes: “Yes” = infection with 1 or more pathogens of the specified category. “No” = no infections of the specified category. Lactulose, Rhamnose and L:R ratio expressed as medians (q1,q3)

**S3b Table:** Agreement between pathogens detected on TAC vs H & E stained microscopy

| Giardia histopathology | Giardia TAC | | Total |
| --- | --- | --- | --- |
|  | Neg | Pos |  |
| Neg | 21 | 16 | 37 |
| Pos | 1 | 22 | 23 |
| Total | 22 | 38 | 60 |

**Giardia diagnosed on TAC vs duodenal histopathology**

There is a significant agreement between TAC and histopathology scores: McNemar p-value < 0.001.

**H pylori diagnosed on TAC vs gastric histopathology**

| H pylori histopathology | H pylori TAC | | Total |
| --- | --- | --- | --- |
|  | Neg | Pos |  |
| Neg | 31 | 1 | 32 |
| Pos | 22 | 6 | 28 |
| Total | 53 | 7 | 60 |

There is a significant agreement between TAC and histopathology scores: McNemar p-value < 0.001.
