# Supplementary material for: Gut integrity and duodenal enteropathogen burden in undernourished children with environmental enteric dysfunction
Source: PLoS Negl Trop Dis. 2021 Jul 15;15(7):e0009584. doi: 10.1371/journal.pntd.0009584 (PMC8352064; doi:10.1371/journal.pntd.0009584)
Supplement: S4 Table — (DOCX) [file pntd.0009584.s005.docx]

**S4 Table:** Comparison of demographic characteristics of cohorts from Faubion *etal* study [11] (USA, Peru & Zambia) and Pakistani cohorts

| Variables | USA  (n=27) | Peru  (n=19) | Zambia  (n=85) | Pakistan cases  (n=60) | Pakistani controls  (n=37) |
| --- | --- | --- | --- | --- | --- |
| Age (months), median (range) | 8.0  (2.0 to 13.0) | 27.0  (15.0 to 29.0) | 21.0  (12.0 to 36.0) | 13.66  (10.3 to 12.16) | 14.16  (9.98 to 17.05) |
| Gender, N (%)  F  M | 8 (30)  19 (70) | 8 (42)  11 (58) | 45 (63)  27 (38) | 19 (30.2)  44 (69.8) | 26 (52)  24 (48) |
| HAZ,  median (range) | −0.1  (−1.8 to 2.4) | −1.8  (−3.3to −0.2) | −2.3  (−8.5 to 1.2) | -2.83  (-5.9 to -0.41) | -1.05  (-2.98 to 1.81) |
| WAZ,  median (range) | 0.5  (−0.4 to 2.1) | −0.8  (−2.7 to 0.8) | −1.4  (−4.0 to 1.1) | -3.26  (-5.62 to -1.79) | -0.21  (-2.97 to 1.24) |
| Lactulose (ug/mL) median (range) | 6.78  (0.29 to 31.9) | 47.60  (4.23 to 379) | 75.40  (0.67 to 873) | 27.0  (0.30 to 264) | 38.0  (0.30 to 346) |
| Rhamnose (ug/mL)  median (range) | 50.1  (0.29 to 213) | 102  (2.66 to 301) | 34.5  (1.58 to 847.) | 66.0  (0.50 to 323) | 86.5  (0.6 to 477) |
| L:R ratio  median (range) | 0.14  (0.06 to 1.0) | 0.75  (0.15 to 5.02) | 2.26  (0.08 to 14.48) | 0.47  (0.09 to 3.08) | 0.51  (0.12 to 1.09) |
