# Supplementary material for: Gut integrity and duodenal enteropathogen burden in undernourished children with environmental enteric dysfunction
Source: PLoS Negl Trop Dis. 2021 Jul 15;15(7):e0009584. doi: 10.1371/journal.pntd.0009584 (PMC8352064; doi:10.1371/journal.pntd.0009584)
Supplement: S5 Table — (DOCX) [file pntd.0009584.s006.docx]

**S5 Table:** Sub-group analysis of dual sugars to gain further insight.

| Variable |  | **True controls** | **Controls** | **Biopsy Cases** |
| --- | --- | --- | --- | --- |
|  |  | N=20 | N=30 | N=63 |
| WAZ mean ±SD | at 6 mo | 0.11 ±1.19 | 0.11 ±0.72 | -3.56 ±1.04 |
|  | at 24 mo | -1.17 ±0.89 | -0.84 ±1.03 | -2.96 ±0.84 |
| WHZ mean ±SD | at 6 mo | 1.07 ±1.07 | 0.67 ±0.79 | -2.89 ±0.82 |
|  | at 24 mo | -0.34 ±0.85 | -0.13 ±0.74 | -1.92 ±0.80 |
| HAZ mean ±SD | at 6 mo | -1.02 ±1.05 | -0.55 ±0.79 | -2.27 ±1.14 |
|  | at 24 mo | -1.63 ±0.95 | -1.30 ±1.28 | -2.90 ±1.14 |
| samples available for analysis (n) | | 14 | 23 | 60 |
| Lactulose µg/ml [median (Q1, Q3)] | | 25.5 (3.9, 82.7) | 39.5 (13.0, 61.0) | 27.0 (11.5,59.5) |
| Rhamnose µg/ml [median (Q1, Q3)] | | 104.5 (5.5, 216.5) | 86.5 (62.0, 182.5) | 60.0 (28.0,178.0) |
| L:R ratio [median (Q1, Q3)] | | 0.47 (0.32, 0.77) | 0.52 (0.30, 0.66) | 0.47 (0.24, 0.90) |

Difference between the dual sugars and their ratio was non-significant among the three groups.
