# Supplementary material for: Gut integrity and duodenal enteropathogen burden in undernourished children with environmental enteric dysfunction
Source: PLoS Negl Trop Dis. 2021 Jul 15;15(7):e0009584. doi: 10.1371/journal.pntd.0009584 (PMC8352064; doi:10.1371/journal.pntd.0009584)
Supplement: S6 Table — (DOCX) [file pntd.0009584.s007.docx]

**S6A Table:** Comparison of dual sugar urinary excretion between different age groups

1. Biopsy cases

| Age at the time of dual assay | <15 months | ≥15 months | p-value |
| --- | --- | --- | --- |
| n | 41 | 19 |  |
| Lactulose, median (IQR) | 20.0 (11.0, 48.0) | 29.0 (15.0, 66.0) | 0.41 |
| Rhamnose, median (IQR) | 52.0 (22.0, 128.0) | 95.0 (34.5, 208.5) | 0.19 |
| LR_ratio, median (IQR) | 0.48 (0.26, 0.87) | 0.40 (0.25, 0.94) | 0.68 |

1. Controls

| Age at the time of dual assay | <15 months | ≥15 months | p-value |
| --- | --- | --- | --- |
| n | 21 | 16 |  |
| Lactulose, median (IQR) | 41.5 (20.5, 65.5) | 25.5 (3.7, 50.0) | 0.11 |
| Rhamnose, median (IQR) | 91.0 (43.0, 163.0) | 83.0 (5.9, 233.0) | 0.70 |
| LR_ratio, median (IQR) | 0.52 (0.38, 0.65) | 0.41 (0.30, 0.71) | 0.76 |

**S6B Table:** Comparison of dual sugar urinary excretion between the genders

1. Biopsy cases

| Gender: | Male | Female | p-value |
| --- | --- | --- | --- |
| N | 44 | 19 |  |
| Lactulose, median (IQR) | 27.5 (12.0, 66.0) | 24.50 (11.0, 48.0) | 0.96 |
| Rhamnose, median (IQR) | 80.0 (29.0, 178.0) | 47.50 (22.0, 128.0) | 0.41 |
| LR_ratio, median (IQR) | 0.42 (0.23, 0.88) | 0.57 (0.37, 1.03) | 0.19 |

1. Controls

| Gender: | Male | Female | p-value |
| --- | --- | --- | --- |
| N | 26 | 24 |  |
| Lactulose, median (IQR) | 39.5 (15.5, 57.5) | 36.0 (7.80, 70.0) | 0.75 |
| Rhamnose, median (IQR) | 86.0 (56.0, 211.0) | 87.0 (12.00, 156.0) | 0.61 |
| LR_ratio, median (IQR) | 0.52 (0.26, 0.71) | 0.50 (0.33, 0.65) | 0.91 |
