# Supplementary material for: Gut integrity and duodenal enteropathogen burden in undernourished children with environmental enteric dysfunction
Source: PLoS Negl Trop Dis. 2021 Jul 15;15(7):e0009584. doi: 10.1371/journal.pntd.0009584 (PMC8352064; doi:10.1371/journal.pntd.0009584)
Supplement: S7 Table — Statistical comparison between each pathogen group vs no pathogen (A) and their spearman correlation (B). (DOCX) [file pntd.0009584.s008.docx]

**S7 Table:** Statistical comparison between each pathogen group vs no pathogen (**A**) and their spearman correlation (**B**).

1. Summary of pathogen groups

| Type of Pathogen | n | % |
| --- | --- | --- |
| **None** | **9** | **15** |
| Bacteria | 12 | 20 |
| Any pathogen, no Bacteria | 39 | 65 |
|  |  |  |
| Protozoa | 40 | 66.67 |
| Any pathogen, no Protozoa | 11 | 18.33 |
|  |  |  |
| Viruses | 9 | 15 |
| Any pathogen, no Viruses | 42 | 70 |

1. Spearman correlation between presence of number of any pathogens, bacteria, protozoa, and viruses (n=60)

|  | Presence of any pathogen | Presence of Bacteria | Presence of Protozoa |
| --- | --- | --- | --- |
| Presence of Bacteria | 0.623 (<.001) |  |  |
| Presence of Protozoa | 0.625 (<.001) | 0.284 (0.028) |  |
| Presence of Viruses | 0.267 (0.040) | -0.057 (0.663) | 0.003 (0.983) |
