# Supplementary material for: Gut integrity and duodenal enteropathogen burden in undernourished children with environmental enteric dysfunction
Source: PLoS Negl Trop Dis. 2021 Jul 15;15(7):e0009584. doi: 10.1371/journal.pntd.0009584 (PMC8352064; doi:10.1371/journal.pntd.0009584)
Supplement: S8 Table — (DOCX) [file pntd.0009584.s009.docx]

**S8 Table:** Microscopic features of gastric, sigmoidal and duodenal biopsies from cases.

1. Summary of histopathological findings on gastric biopsies

| Clinical diagnosis on Gastric microscopy | Pathogen | |
| --- | --- | --- |
|  | With H pylori | Without H pylori |
| Normal gastric tissue (n) | 0 | 3 |
| Mild non-specific gastritis (n) | 10 | 24 |
| Mild chronic gastritis (n) | 5 | 4 |
| Moderate non-specific gastritis (n) | 13 | 3 |
| Severe gastritis (n) | 1 | 0 |
| **Total** | **29** | **34** |

1. Summary of histopathological findings on sigmoidal biopsies

| Clinical diagnosis on Sigmoidal microscopy | (n) |
| --- | --- |
| Mild sigmoiditis with eosinophils | 3 |
| Mild non-specific colitis | 7 |
| Mild lymphocytic infiltrations | 2 |
| Moderate active sigmoiditis | 4 |
| Moderate non-specific sigmoiditis | 1 |
| **Total** | **17** |

1. Summary of histopathological findings on rectal biopsies

| Clinical diagnosis on Rectal microscopy | (n) |
| --- | --- |
| Mild focal colitis | 3 |
| Mild non-specific proctitis | 7 |
| Mild chronic colitis | 1 |
| Moderate active proctitis | 4 |
| Moderate non-specific proctitis | 5 |
| **Total** | **20** |
